# Calcifying fibrous tumor presenting as rectal submucosal tumor: first case reported in rectum

**DOI:** 10.1186/1477-7819-12-28

**Published:** 2014-02-03

**Authors:** Soyoung Im, Ji-Han Jung, Changyoung Yoo, Hyun Joo Choi, Jinyoung Yoo, Chang Suk Kang

**Affiliations:** 1 Department of Hospital Pathology, St. Vincent’s Hospital, The Catholic University of Korea, 93, Ji-dong, Paldal-gu, Suwon 442-723, Republic of Korea; 2 Department of Hospital Pathology, Yeouido St. Mary’s Hospital, The Catholic University of Korea, Seoul, Republic of Korea

**Keywords:** Calcifying fibrous tumor, Submucosal tumor, Rectum

## Abstract

Calcifying fibrous tumor (CFT) is a recently recognized rare benign lesion characterized by dense hyalinized collagenous tissue with interspersed spindle cells and a lymphoplasmocytic infiltrate. Calcification is the hallmark of CFT and may present in the form of psammomatous bodies or dystrophic calcifications. CFT of the intestinal tract is uncommon and rectal CFT has never been reported. Recently, we experienced a case of CFT found in the rectum of a 36-year-old man. In this study, we described the characteristic histopathological findings with a review of the relevant literature. Although CFT of the intestinal tract as an intrinsic visceral lesion is unusual and clinically unexpected, CFT should be considered in the differential diagnosis of rectal submucosal tumor.

## Background

Calcifying fibrous tumor (CFT) is a rare, benign soft-tissue tumor of unknown etiology, characterized by the presence of hyalinized collagenous fibrous tissue with psammomatous or dystrophic calcification and focal lymphoplasmocytic infiltrate [1]. These lesions were originally described as soft tissues of the extremities, trunk, neck, or axilla [1]. In addition, subsequent sporadic reports revealed that CFT can also arise from pleura, mediastinum, peritoneum, mesentery, and lung [2-5]. However, CFTs of the intestinal tract as an intrinsic visceral lesion are quite rare [6-8]. To the best of our knowledge, CFT of the rectum has never been previously reported. Here, we present the first case of CFT with metaplastic ossification of the rectum.

## Case presentation

**Figure 1:**
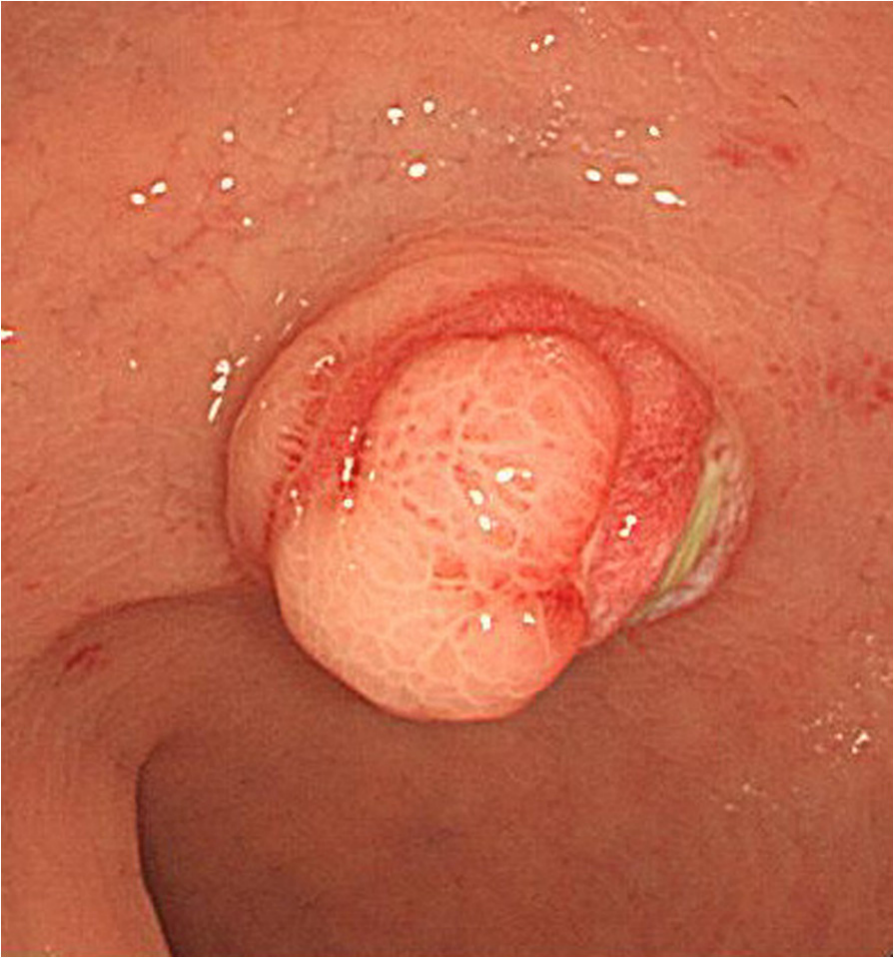
Colonoscopic finding shows an ovoid polypoid mass with normal overlying mucosa in the proximal rectum.

**Figure 2:**
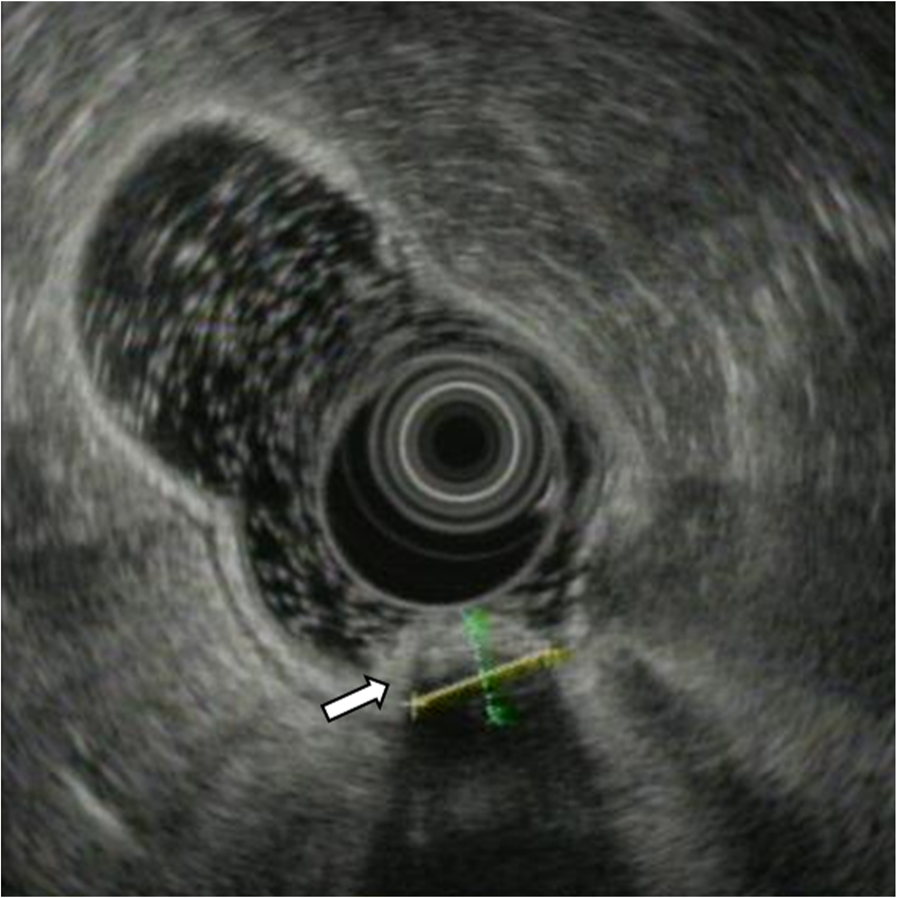
Endoscopic ultrasonographic finding shows a well-demarcated submucosal mass (arrow).

**Figure 3:**
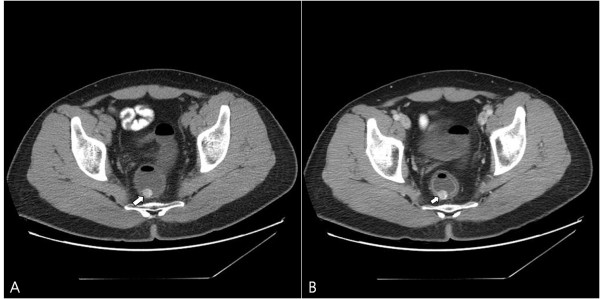
Plain (A) and enhanced (B) computed tomography of the abdomen reveals a prominent contrast-enhancing rectal wall mass with internal calcification (arrow).

**Figure 4:**
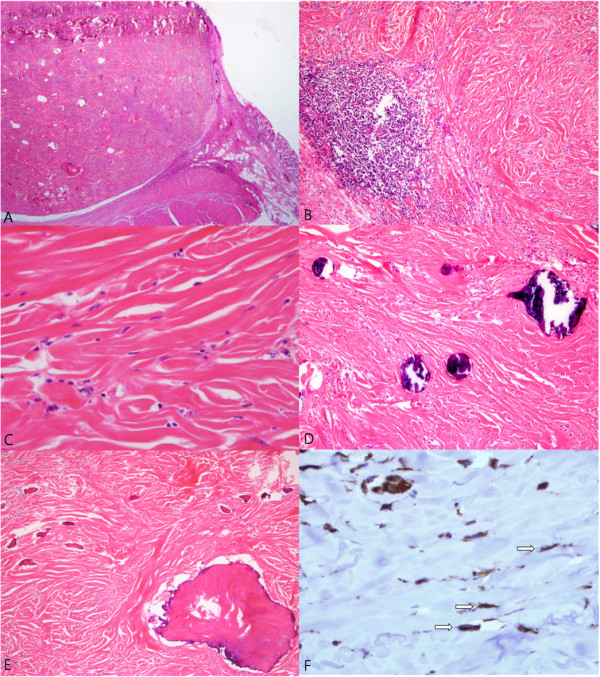
**Microscopic findings of the tumor. (A)** Whole mount of the well-circumscribed tumor located in the submucosa (H&E x1.4). **(B)** Microscopic finding of the tumor shows dense fibrous tissue and lymphoid aggregates (H&E x100). **(C)** The spindle cells have bland nuclei and indistinct cytoplasm (H&E x400). **(D)**Psammomatous or dystrophic calcifications are seen (H&E x100). **(E)** Metaplastic ossifications are also noted (H&E x100). **(F)** Immunohistochemical staining for vimentin shows diffuse positive for tumor cells (arrow) (x400).

A 36-year-old man was referred to our hospital because of a rectal polyp found incidentally by colonoscopy at another hospital. He had no significant past medical history. Family history was non-specific. The physical examination including an abdominal examination was normal. Hemoglobin and hematocrit, chemistry panel with a liver function test, and urine analysis were normal. A full colonoscopy revealed a 1.8 cm-sized ovoid polypoid mass with firm consistency and normal overlying mucosa seen 10.0 cm from anal verge (Figure 1). The mass, as viewed by endoscopic ultrasonography, appeared as a well-circumscribed submucosal tumor with mixed hyperechoic and hypoechoic patterns (Figure 2). The carcinoembryonic antigen serum level was normal. Computed tomography of the abdomen revealed a prominent contrast-enhancing rectal wall mass with internal calcification, and there were no remarkable perirectal tumor infiltrates or lymphadenopathy (Figure 3). Gastrointestinal stromal tumor or leiomyoma was suspected both radiologically and endoscopically. Preoperative biopsy of the mass was not performed. Instead, the endoscopist tried to remove the mass by endoscopic submucosal resection. However, it was unsuccessful due to its hardness and so transanal local excision was performed. On gross examination, the resected specimen revealed a 1.8 cm, well-circumscribed but unencapsulated, gray-white, firm rectal mass. Microscopically, the tumor was located in the submucosa and created surface ulceration (Figure 4A). The tumor consisted of sparsely cellular, collagenous, fibrous tissue and multiple aggregates of inflammatory cells including lymphocytes and plasma cells (Figure 4B). Uniform, spindle-shaped tumor cells were dispersed among thick collagenous bundles (Figure 4C). Some psammomatous and dystrophic calcification and metaplastic ossification were noted (Figure 4D and 4E). There were no areas of tumor necrosis, cellular anaplasia, abnormal mitosis, or any features of malignancy present. Immunohistochemical staining showed that the spindle cells were diffusely positive for vimentin (Figure 4F) and were negative for CD117 (c-kit), CD34, actin, desmin, S-100 protein, nuclear β-catenin, and anaplastic lymphoma kinase (ALK). The histology was consistent with a benign, calcifying fibrous tumor.

CFT is a rare benign soft-tissue tumor originally described by Rosenthal and Abdul-Karim [9] as a childhood fibrous tumor with psammoma bodies in two- and eleven-years-old girls. Fetsch *et al*. [1] reported ten similar subcutaneous and visceral soft tissue lesions and first used the term calcifying fibrous pseudotumor (CFP). However, Nascimento *et al*. [10] found that three of ten patients of CFPs with available follow-up showed a local recurrence and proposed that CFP is a distinctive benign mesenchymal neoplasm with a local recurrence and therefore, best renamed ‘calcifying fibrous tumor (CFT)’. Histologically, this lesion is characteristic; a heavily collagenized paucicellular fibrous lesion composed of bland spindle cells, scattered psammomatous and/or dystrophic calcification, and variably prominent mononuclear inflammatory infiltrate [1,9-11]. Unusually, metaplastic ossification was also reported [12]. Immunochemically, the spindle cells of CFTs express vimentin and factor XIIIa, but usually lack actin, desmin, S-100 protein, CD34, and ALK-1 [12,13]. Clinically, CFTs show a predilection for children and young adults [9], and mainly arise from soft tissues of the extremities, trunk, neck, groin, and axilla [1]. More recently, several cases have presented as an intra-abdominal CFT in the peritoneum and abdominal cavities, suggesting that the serosa is the favored site of origin of intra-abdominal CFT [3,14]. However, CFTs of intestinal tract, especially as an intrinsic visceral lesion, are extremely rare. We identified five cases of intestinal CFT reported in the relevant English medical literature (Table 1). To the best of our knowledge, CFT of the rectum has never been reported. These six cases, including the present case, arose in four men and two women, with a mean patient age of 30 years (range, 20 to 38 years) and tended to be smaller (mean 1.7 cm). Involved organs were the ileum (n = 4), colon (hepatic flexure) (n = 1), and rectum (n = 1). Three cases were subserosal, two cases centered in the submucosa, and one case was localized within the muscle layer. Their characteristics including male predominance (66%), smaller tumor size, and unifocal occurrence contrast with no gender predilection and relatively larger tumor size of soft tissue CFTs [11], and with female predominance (70%) and common multifocality of their peritoneal counterparts [3,11], suggesting different pathogenetic pathways regardless of morphologic similarity.

**Table 1:** Review of literature about intestinal calcifying fibrous tumors (CFTs)

**Case**	**Reference**	**Age/sex**	**Site**	**Type**	**Size(cm)**	**Multiple**	**Clinical presentation**
1	Emanuel *et al.*[6]	20/M	Ileum	Intramural	2.0	No	Obstruction, Intussusception
2	Emanuel *et al.*[6]	38/F	Ileum	Subserosal	3.3	No	Abdominal pain
3	Emanuel *et al.*[6]	30/F	Ileum	Subserosal	0.5	No	Incidental
4	Emanuel *et al.*[6]	35/M	Ileum	Subserosal	0.5	No	Incidental
5	Shi *et al.*[7]	22/M	Colon	submucosal	2.0	No	Abdominal discomfort
6	Present case	36/M	Rectum	submucosal	1.8	No	Incidental

Both the cause and pathogenesis of CFTs are unclear. A few studies reported single or multiple tumor masses that showed admixed histopathologic features of both CFT and inflammatory myofibroblastic tumor (IMT) and suggested CFT might represent a late sclerosing stage of IMT [15-17]. Conversely, there have been studies demonstrating that the CFTs have different histological, immunohistochemical, and electron microscopic features from IMTs, suggesting that CFP and IMT are distinct lesions [10,13,18]. Further study about the pathogenesis of CFT, especially intestinal CFT is needed.

The endoscopical and radiological differential diagnosis of intestinal CFT presenting as submucosal spindle cell tumor includes gastrointestinal stromal tumor (GIST), leiomyoma, schwannoma, inflammatory fibroid polyp (IFP), desmoid-type fibromatosis, and IMT. Recently, endoscopic ultrasound (EUS)-guided fine needle aspiration (FNA) has emerged for diagnostic sampling of gastrointestinal subepithelial lesions to enhance the diagnosis compared to using EUS imaging alone [19]. Watson *et al*. [20] retrospectively reviewed 66 cases of submucosal tumors which underwent EUS-FNA with IHC and consecutive surgical resection. According to their report, cytology results from EUS-FNA were either diagnostic (68%) or suspicious (12%) in a total of 80%. Therefore, EUS-FNA with immunohistochemistry studies was one of the most useful tools for differentiating various gastrointestinal submucosal tumors. However, EUS-FNA was not performed in this case. As regards pathological differential diagnosis, although GIST and smooth muscle tumor may have degenerative or regressive changes including fibrosis, hyalinization, and calcification, psammomatous calcification and lymphoplasmocytic infiltrates are not features of GIST and smooth muscle tumor [11]. Moreover, immunohistochemical stainings for CD117, CD34, actin, and desmin in CFTs were usually negative. The presence of peritumoral or intratumoral lymphoid aggregates may raise the possibility of schwannoma, but this was ruled out by absence of residual schwannoma tissue and a complete lack of S-100 reactivity. Owing to focal CD34 reactivity in abdominoperitoneal CFTs, IFP should be considered [21]. However, most CFTs lack cellularity and the onion-skin pattern of IFP. Desmoid-type fibromatosis is a locally aggressive fibrogenic neoplasm that mainly arises in the small intestine mesentery. In contrast to CFTs, the lesions lack the psammomatous or dystrophic calcification, have an infiltrative border, are positive for nuclear β-catenin, and are negative for factor XIIIa. IMT is a relatively well-defined disease entity characterized by proliferation of myofibroblastic spindle cells with an inflammatory cell infiltrate. In addition, areas with sclerosis and relatively sparse inflammatory cells, and coarse or psammomatous calcification are occasionally seen IMT [11,17]. However, large, amorphous calcified areas are not a feature of IMT. Immunohistochemically, IMTs are positive for actin and ALK-1, while only focally positive for factor XIIIa [6,11,12,17]. In our case, the typical histopathological features that are similar to their soft tissue counterparts and negative immunohistochemical staining results for CD117, CD34, actin, desmin, β-catenin, S-100 protein, and ALK-1, allow a diagnosis of CFT of rectum. In addition, metaplastic ossification was noted; an unusual finding, only one case of which has been previously reported [12].

The prognosis of CFT remains good in most cases. However, local recurrence after complete or incomplete excision has been reported; the rate of recurrence varies from 17% to 30% in studies with the largest series of CFTs [1,10]. So, the treatment of choice for CFT is complete local excision with clear margins [7,17]. In the present case, no recurrence was observed during eight months follow-up; however this follow-up period is too short and although recurrence was not mentioned in other cases of intestinal CFT, careful follow-up remains necessary to detect infrequent and delayed recurrence.

## Conclusions

We herein reported the first case of CFT presenting as a rectal submucosal tumor. Intestinal CFT is a rare entity and is probably under-recognized. Awareness of the typical histological and immunohistochemical features of CFTs will help to distinguish them from other intestinal spindle cell tumors.

## Consent

Written informed consent was obtained from the patient for publication of this case report and any accompanying images. A copy of written consent is available for review by the Editor-in-Chief of this journal.

## Abbreviations

ALK: anaplastic lymphoma kinase; CFP: calcifying fibrous pseudotumor; CFT: calcifying fibrous tumor; GIST: gastrointestinal stromal tumor; IFP: inflammatory fibroid polyp; IMT: inflammatory myofibroblastic tumor.

## Competing interests

The authors declare that they have no competing interests.

## Authors’ contributions

SI and JJ conceived of the study, collected data and drafted the manuscript. CY performed histological and immunohistochemistry evaluation. HC participated in the collecting and editing of images. JY helped in drafting the manuscript. CK corrected and revised the manuscript. All authors read and approved the final manuscript.
